# Does the nurse-led case management benefit rheumatoid arthritis patients in reducing distressing symptoms and C-reactive protein: a 2-year follow-up study in Taiwan

**DOI:** 10.3389/fmed.2024.1373639

**Published:** 2024-06-06

**Authors:** Wei-Chiao Chang, Hanoch Livneh, Hua-Lung Huang, Hsin-Hua Li, Ming-Chi Lu, Miao-Chiu Lin, Wei-Jen Chen, Tzung-Yi Tsai

**Affiliations:** ^1^Department of Chinese Medicine, Dalin Tzu Chi Hospital, Buddhist Tzu Chi Medical Foundation, Chiayi, Taiwan; ^2^Rehabilitation Counseling Program, Portland State University, Portland, OR, United States; ^3^Department of Rehabilitation, Dalin Tzu Chi Hospital, Buddhist Tzu Chi Medical Foundation, Chiayi, Taiwan; ^4^Division of Allergy, Immunology and Rheumatology, Dalin Tzu chi Hospital, Buddhist Tzu chi Medical Foundation, Chiayi, Taiwan; ^5^School of Medicine, Tzu Chi University, Hualien, Taiwan; ^6^Department of Nursing, Dalin Tzu Chi Hospital, Buddhist Tzu Chi Medical Foundation, Chiayi, Taiwan; ^7^Graduate Institute of Sports Science, National Taiwan Sport University, Taoyuan, Taiwan; ^8^School of Post-Baccalaureate Chinese Medicine, Tzu Chi University, Hualien, Taiwan; ^9^Center of Sports Medicine, Dalin Tzu Chi Hospital, Buddhist Tzu Chi Medical Foundation, Chiayi, Taiwan; ^10^Department of Environmental and Occupational Health, College of Medicine, National Cheng Kung University, Tainan, Taiwan; ^11^Department of Nursing, Tzu Chi University of Science and Technology, Hualien, Taiwan; ^12^Department of Medical Research, Dalin Tzu Chi Hospital, Buddhist Tzu Chi Medical Foundation, Chiayi, Taiwan

**Keywords:** fatigue, generalized estimating equations, C-reactive protein, nurse-led case management, pain, rheumatoid arthritis

## Abstract

**Background:**

Rheumatoid arthritis (RA) is a chronic disease and may worsen over time. Today, nurse-led case management (NLCM) has been recommended to improve clinical outcomes for chronic disease patients, yet little is known regarding its impact on pain, fatigue, and C-reactive protein (CRP) among RA patients. We aimed to explore this issue among such groups via a two-group pre- and post-test approach.

**Methods:**

All subjects were recruited from one hospital in Taiwan from January 2017 to June 2018 and assigned to either a 6-month NLCM program in addition to usual care or to a control group that received usual care only. All of them were followed for 2 years. Outcomes of interests were compared at four time points: baseline, the third day after NLCM completion, and at 6 and 24 months after NLCM. Effects between them were tested using the generalized estimating equations (GEE) model after adjusting for differences at baseline.

**Results:**

A total of 50 patients in the NLCM group and 46 in the control group were recruited for data analysis. Results from the GEE model indicated that integrating NLCM into conventional care benefited patients in decreasing levels of pain and fatigue, as well as CRP value. These improvements were still observed for 2 years after NLCM.

**Conclusion:**

NLCM was shown to be helpful in lowering pain, fatigue, and CRP, which implies that NLCM may be a reference in the provision of tailored care for those affected by rheumatism.

## Introduction

Rheumatoid arthritis (RA), an autoimmune disease with systemic inflammation, can cause a wide range of uncomfortable symptoms, from mild joint stiffness to severe functional disability. According to the National Health Interview Survey in 2011–2013, arthritis/rheumatism was one of the top three leading causes of long-term disability in the United States, causing a tremendous socioeconomic burden (1). A nationwide estimation in the United States showed that annual direct medical costs of newly diagnosed RA were $20,919 per patient on average, which nearly tripled that of those without RA ($7,197) (2).

Beyond joint pathology, RA may place people at a higher risk of developing other extra-articular manifestations due to systemic inflammation, such as cardiovascular disease, pulmonary disease, or cancer (3, 4), thereby leading to a higher mortality rate than the general population (5). In such a case, *a priori* study that followed patients with inflammatory arthritis had found that C-reactive protein (CRP), a commonly used marker of systemic inflammation in RA (6), was an independent predictor of mortality from cardiovascular diseases among this group (7). Thus, in view of the irreversible nature of RA, some specific distressing side effects, such as fatigue and pain, maybe the prevalent symptoms associated with this illness. It has been estimated that one in every two RA patients may experience persistently high levels of fatigue or pain (8, 9). Making matters worse, fatigue not only increased the length of hospitalization by 83% (10) but also caused a conspicuous increase in the likelihood of mortality (11). Therefore, actively implementing a disease management program to minimize the distressing symptoms and inflammation status driven by RA is of paramount importance.

Case management is a widely used care model for patients with chronic diseases who require consistent management over prolonged periods. Specifically, case management represents “a collaborative process of assessment, planning, facilitation, care coordination, evaluation, and advocacy for options and services to meet an individual’s and family’s comprehensive health needs through communication and available resources to promote quality, cost-effective outcomes” (12, 13). In this model, a specialized nurse often takes on the primary coordination, management, and continuity of care for a specific episode of treatment or intervention (14). Accordingly, the European League Against Rheumatism has put out a call on the integration of case management into routine care to meet quality-of-life needs and to assist with the management of RA symptoms (15). Recently, the use of nurse-led case management (NLCM) for RA patients has attracted a lot of attention, but there is a lack of consensus regarding its impacts. Several studies addressed that the NLCM group experienced remarkable increases in self-efficacy ability and disease activity scores, which were measured by a 28-joint scale (DAS 28) (16–20). Another study also showed that NLCM minimized the RA patients’ daily disability assessed by the Health Assessment Questionnaire-Disability Index (21). However, some investigations failed to reveal a significant association between NLCM and DAS 28 scores among RA patients (18, 22–24). The controversy over the effectiveness of NLCM may be related to the neglect of the cluster-specific baseline adjustments and the potential auto-correlations within subjects across time in the priori research, thus prejudicing the findings.

Furthermore, as of now, most of the relevant assessments of NLCM were performed in Western populations (8, 19, 21, 22, 24). The direct application of the results to Chinese populations may be premature because of the intrinsic differences in lifestyle and environmental features between Chinese and Western populations. A noteworthy feature of the priori evidence is that there is a lack of knowledge of the long-term NLCM effects among RA patients, especially in changes of pain, fatigue, and inflammatory symptomatology. To fill the gap, we carried out a study that followed RA participants until a 2-year period following completion of the NCLM program to compare the changes over time in both their subjective symptoms and inflammation status. Such documentation could provide an empirically robust ground for healthcare policymakers to initiate more appropriate care processes for individuals with RA.

## Methods

### Design and participants

First, this non-randomized follow-up study enrolled RA participants from a rheumatological clinic of the target hospital in Taiwan from January 2017 to June 2018, and all patients were followed for 2 years. To be eligible, the subjects were required to have a diagnosis of RA by rheumatologists, utilizing the classification criteria published by the 2010 American College of Rheumatology and the European League Against Rheumatism (EULAR) (25, 26), and aged 20 years or older at the time of recruitment. Those unable to reliably express their opinions or sign a written consent were excluded. Additionally, we marked all questionnaires with an encryption code instead of any personal identifiers. The required sample size in this study was determined based on previous research, where the effect size was set at 0.33, which concentrated on the fatigue change between the two groups (22), and the power was set to 80% at a significant level at 0.05. Hence, at least 92 participants were required for reliable statistical calculation using PASS 14.0 software (NCSS, Kaysville, UT, USA).

Before participating in the present research, all enrollees received detailed written and verbal information about the aims and protocol of the present study and signed informed consent. Participants were free to withdraw from the study at any time without any penalty. The current study protocol has been approved by the Institutional Review Board and the ethics committee of the target hospital (No. B11004003). The enrollees were informed that all personal information would be kept confidential.

### Procedure

**Figure 1:**
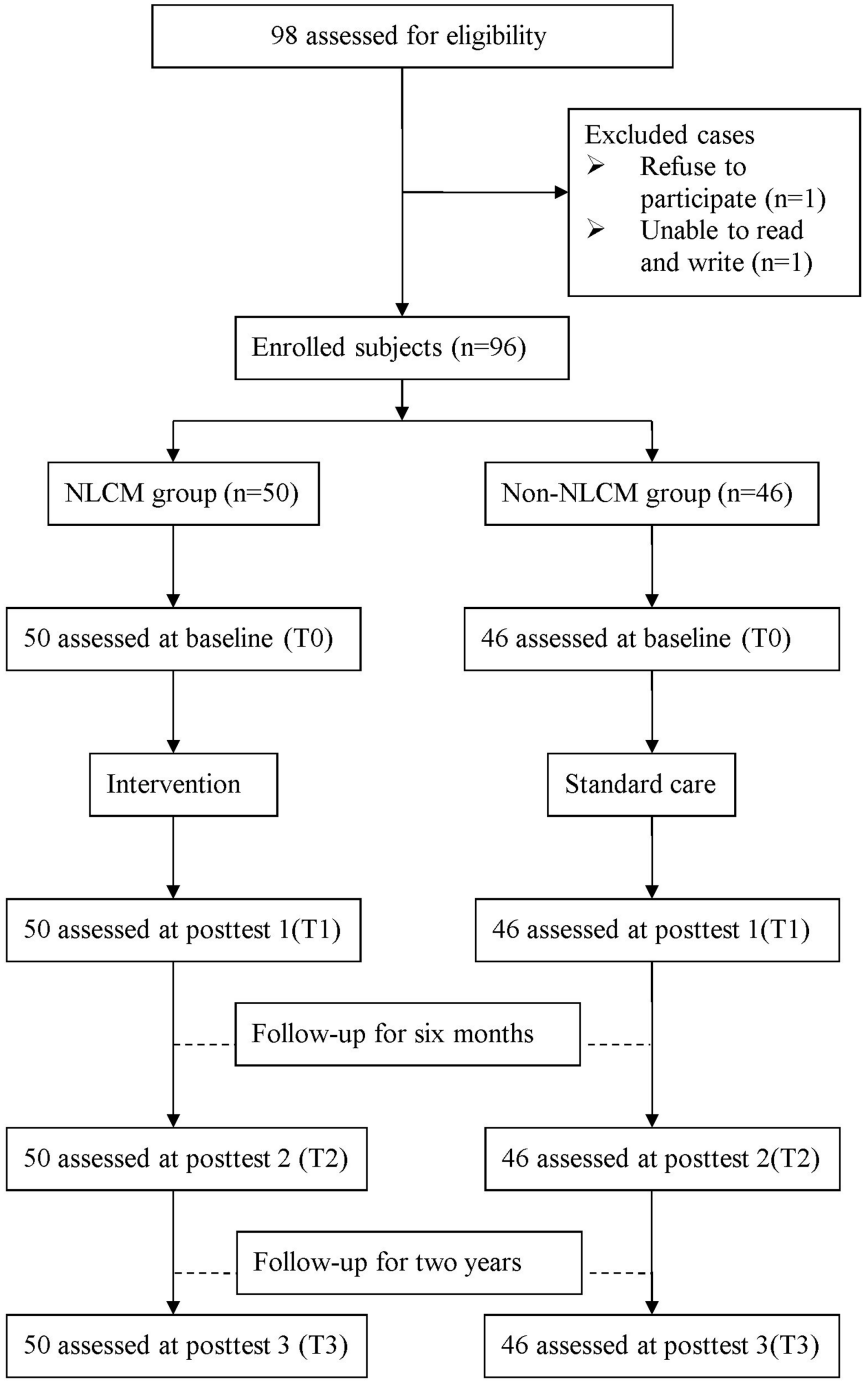
Participant recruitment flowchart.

A flowchart of the participant recruitment is displayed in Figure 1. Before the study commenced, all participants were instructed to complete a form that included information on socioeconomic status, prescription medications, and self-health management behaviors. In addition, the information on the outcome indicators was assessed at four time points: baseline (T0), 3 days after completion of NLCM (T1), 6 months after NLCM completion (T2), and 24 months after completion of NLCM (T3). One independent interviewer, who was blinded to the study group assignment, took responsibility for obtaining informed consent and administering all measures during the study timeframe. To minimize the dropout rate, all enrollees were telephoned and reminded to return to the hospital for the completion of assessments as scheduled.

### Intervention

Patients with RA seeking care in the target hospital were referred to the following process. First, they were informed of their right to opt to receive either conventional care or conventional care plus NLCM. Those joining the conventional care would receive health education lasting for 15 min per medical visit from ward nurses as scheduled, consisting of consultation in terms of disease symptoms, related treatments, and the doctor’s orders. In this study, they would be deemed the non-NLCM group.

By contrast, those enrolling in the NLCM group would receive the consecutive intervention programs that were developed by an extensive review of the literature and discussions with experts. The NLCM consisted of three key components: (1) a series of RA-related education sessions, including disease etiology, complications, and self-care management; (2) instructions on the individual exercise program, containing explanations and demonstrations of the various steps; and (3) monthly telephone follow-up evaluation to identify difficulties faced by the participants, monitor their daily practice, and answer any questions. The first two components were delivered through one-to-one health education sessions lasting for approximately 50 min each, once a month for 6 consecutive months. An educational booklet on the relevant issues discussed was also given to each participant as a guide. Additionally, they were instructed to report time spent on and frequency of exercise per week using the electronic diary provided at a freeware instant-communications app LINE. As a whole, we used an interactive learning environment to allow enrollees to discuss the impact of illness, the treatment received, and changes in self-image and relationships with friends and family. All involved strategies were systematically planned and modified on the basis of the individualized therapeutic regimen offered to the patient. The nursing case manager offered consultation with patient’s family and other healthcare providers of the medical team as needed.

Collectively, the NLCM in the target hospital integrated a multi-component intervention comprised of health education and professional advice, referring patients to other healthcare team members, discussing a daily life plan, making medical appointments, and conducting telephone follow-up provided by the trained registered nurse. This NLCM program was conducted in one rheumatology health education room and delivered by one specific nurse case manager who had more than 10 years of experience in nursing care for RA patients and possessed the NLCM certification.

### Outcome indicators

In this study, we collected three primary outcome indicators, including fatigue, pain, and CRP. These outcome indicators were measured before and after the initiation of intervention, all of which were obtained from patient’s medical records.

In the target hospital, levels of fatigue and pain were both determined via the self-reported Visual Analog Scale (VAS), which has been extensively used and validated across the rheumatic disease spectrum (27, 28). VAS is used to assess the intensity and frequency of subjective pain experienced by the patient on an 11-point numerical scale, ranging from 0 to 10, where higher scores reflect a more severe degree of fatigue or pain experienced (28, 29). The reliability and validity of VAS measures have been previously confirmed (30). The VAS was demonstrated to possess acceptable psychometric properties, with a concurrent validity of 0.90 and an intraclass correlation coefficient of 0.94 (31). In this study, the Cronbach’s alpha scores for pain and fatigue were 0.85 and 0.89, respectively. Apart from these two subjective symptoms, we used CRP as the major marker of inflammation status. CRP is a protein produced by the liver and is a commonly used marker of systemic inflammation for autoimmune diseases (6).

### Covariates

The demographic variables herein included age, sex, marital status, educational level, job status, household status, and lifestyle factors (smoking and exercise habits). Patients who answered “currently” or “yes/past” to the question on smoking were classified as smokers. Patients who exercised 3 or more days per week were classified as having regular exercise. Disease characteristics included comorbidities (diabetes mellitus, hypertension, heart disease, or stroke), body mass index, DAS 28, duration of RA, and use of conventional disease-modifying anti-rheumatic drugs (DMARDs) and biological agents. The latter indicator comprised adalimumab, etanercept, infliximab, rituximab, and tocilizumab. The last two indicators were defined as using the relevant drugs for more than 3 months following disease onset.

### Evaluation of data

The descriptive statistics, including the mean, standard deviation (SD), and percentage, were used to describe the distributions. We then compared the distributions of demographic and disease characteristics between the two groups using the t-test and *χ^2^* test as applicable. Additionally, intergroup differences before and after the intervention for each of the outcomes were tested using the generalized estimating equations (GEE) model, a statistical procedure that extends the capabilities of generalized linear models (GLM) for analyzing longitudinal data or other clustered response data (32). Each GEE model produces estimates of the time effect (baseline as the reference category), intervention effect (control group as the reference category), and effect of the interaction term between intervention and time after covariate adjustment (33). The effect of the intervention, as it varies over time, can, then, be confirmed, provided that the interaction term was pronounced. Covariates, where the difference reached statistical significance at baseline, were identified as the control variables in the GEE model. Robust standard errors were selected to calculate the significance of parameter estimates, and the autoregressive first-order working correlation matrix was utilized to adjust for the time effect (33). All analyses were conducted using SPSS 22.0 (Chicago, IL, USA), and all statistical tests were performed at the two-tailed significance level of 0.05.

## Results

### Baseline characteristics

Demographic and clinical characteristics of the study sample are given in Table 1. During the study period, a total of 96 RA patients were recruited, consisting of 50 in the NLCM group and 46 in the non-NLCM group. None of the participants dropped out or were lost to follow-up. The mean (SD) age was 56.6 (10.3) years in the NLCM group and 50.7 (10.8) years in the control group, respectively. Most participants were women and had comorbidities at the time of the study, and 35% of them had been treated with at least one biological agent. Demographic and clinical characteristics between them were comparable at the baseline, except for age and the duration of RA. Compared with the non-NLCM group, subjects in the NLCM group were found to experience higher scores of DAS 28, pain, and fatigue at baseline (all *p* ≤ 0.01).

**Table 1:** Demographic and clinical data by two groups.

Variable	All participants (*N* = 96)		NLCM group (*n* = 50)		Non-NLCM group (*n* = 46)		*p*
	*N*	%	*N*	%	*N*	%	
Sex (female)	79	82.3	38	76.0	41	89.1	0.09
Marital status (married)	81	84.4	44	88.0	37	80.4	0.31
Educational level (≥ 9th grade)	54	56.2	27	54.0	27	58.7	0.64
Household status (Cohabitating)	89	92.7	46	92.0	43	93.5	0.78
Monthly income (≤ 30,000 NTD)	52	54.2	27	54.0	25	54.3	0.97
Regular exercise	34	35.4	24	48.0	18	39.1	0.15
Cigarette smoking	14	14.6	9	18.0	5	10.9	0.32
Conventional DMARDs	79	82.3	42	84.0	37	80.4	0.65
Biological agents use	34	35.0	18	36.0	16	34.8	0.90
Comorbidities	68	71.0	34	68.0	34	73.9	0.52
	Mean	SD	Mean	SD	Mean	SD	
Age (years)	53.80	10.5	56.66	10.4	50.74	10.8	0.01
Disease duration^†^	5.1	4.3	3.7	3.5	6.6	4.5	0.01
Body mass index (Kg/m^2^)	23.7	3.8	23.2	3.4	24.2	4.1	0.21
DAS 28^†^	4.5	1.6	5.0	1.4	3.9	1.5	<0.01
C-reactive protein	1.5	1.2	1.9	1.1	1.0	1.3	0.06
Pain^†^	5.7	2.4	6.9	2.8	4.4	2.5	<0.01
Fatigue^†^	5.2	3.3	6.3	2.9	4.0	3.3	<0.01

^†^Data were expressed as mean ± standard deviation.

NLCM, nurse-led case management; DAS 28, disease activity score measured by a 28 joint-scale; NTD, New Taiwan Dollar.

### Comparison of effect of NLCM versus conventional care

**Figure 2:**
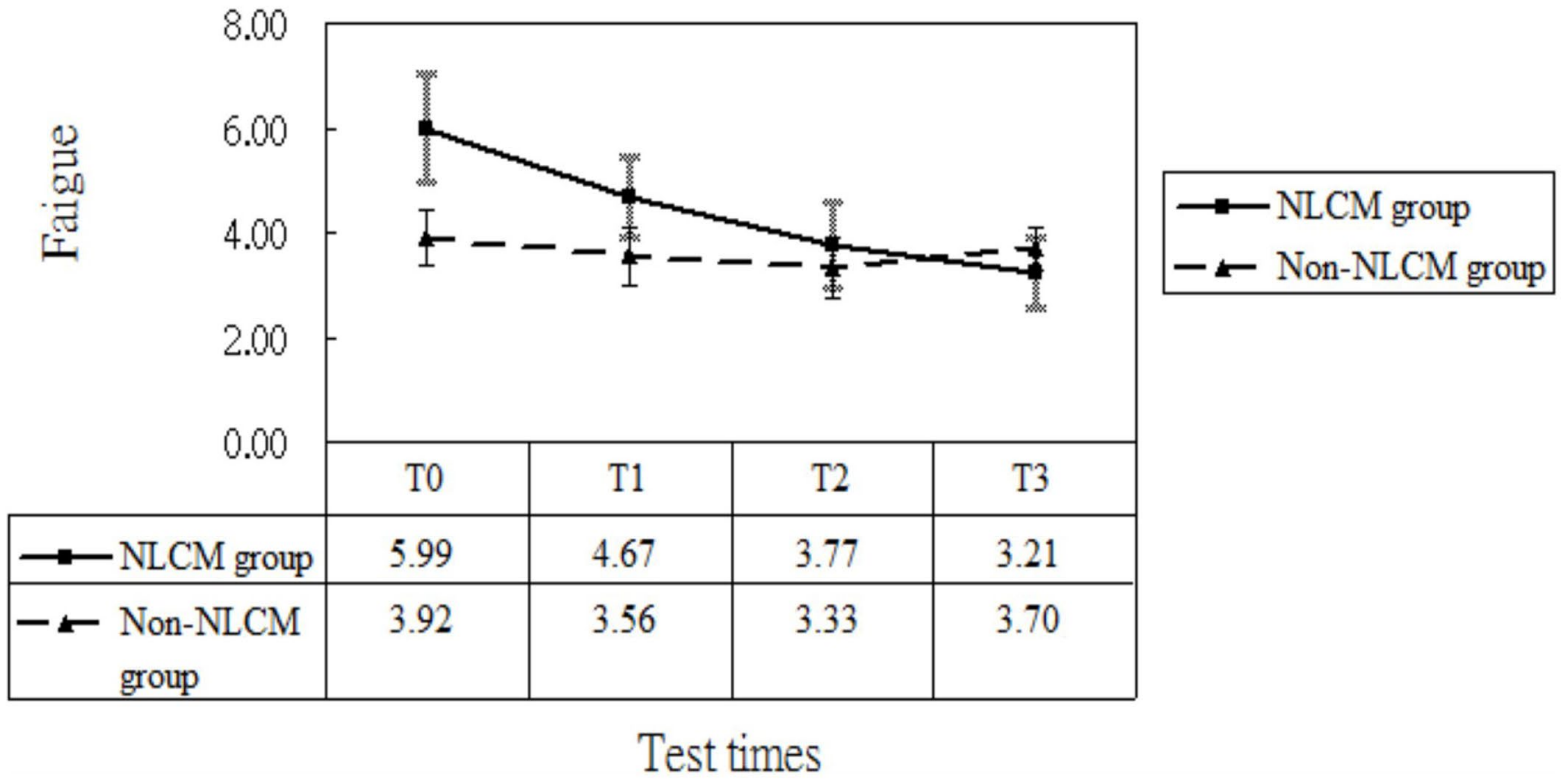
Fatigue scores of patients in the NLCM and the control groups.

After taking into consideration the significant variables at baseline (displayed in Table 1), the multivariate analysis using the GEE procedure indicated that a baseline difference occurred regarding fatigue score between the NLCM and the non-NLCM groups (*p* = 0.03) (Table 2). Fatigue scores at T1, T2, and T3 were similar to those measured at T0, implying that a maturation effect might not have arisen. Following consideration of baseline differences in fatigue, age, DAS 28, and disease duration by GEE procedure, the reduction slope of fatigue scores was still larger in the NLCM group than in the non-NLCM group, irrespective of elapsed time (Figure 2).

**Table 2:** Regression coefficients associated with nurse-led case management (NLCM) on patients with rheumatoid arthritis were obtained by generalized estimating equation model (*n* = 96).

Variables	Fatigue		CRP		Pain	
	*β*	*p*	*β*	*p*	*β*	*p*
Intercept	3.33	<0.01	0.67	0.01	2.56	0.41
NLCM vs. non-NLCM	2.43	0.03	−0.05	0.25	1.54	0.01
Time						
T1 vs. T0	−0.36	0.46	0.98	0.06	−0.66	0.01
T2 vs. T0	−0.59	0.10	1.08	<0.01	−1.05	0.16
T3 vs. T0	−0.22	0.71	0.45	0.02	−1.40	<0.01
Interaction term						
Interaction of T1 × NLCM	−1.32	0.04	−0.66	0.04	−1.29	0.03
Interaction of T2 × NLCM	−1.42	0.02	−0.94	0.01	−1.49	0.02
Interaction of T3 × NLCM	−1.76	<0.01	−0.72	0.03	−1.99	0.01
DAS 28	0.65	<0.01	0.63	0.01	0.90	0.01
Disease duration (year)	−0.04	0.12	−0.03	0.25	−0.02	0.54
Age (year)	−0.02	0.23	0.02	<0.01	0.01	0.56

T0, prior to NLCM inception; T1, the 3th day after NLCM commencement; T2, 6 months after NLCM completion; T3, 2 years after the completion of NCLM. Interaction of T1 × NLCM, difference between NLCM and control group in change from T0 to T1. Interaction of T2 × NLCM, difference between NLCM and control group in change from T0 to T2. Interaction of T3 × NLCM, difference between NLCM and control group in change from T0 to T3. CRP, C-reactive protein; DAS 28, disease activity score measured by a 28-joint scale.

**Figure 3:**
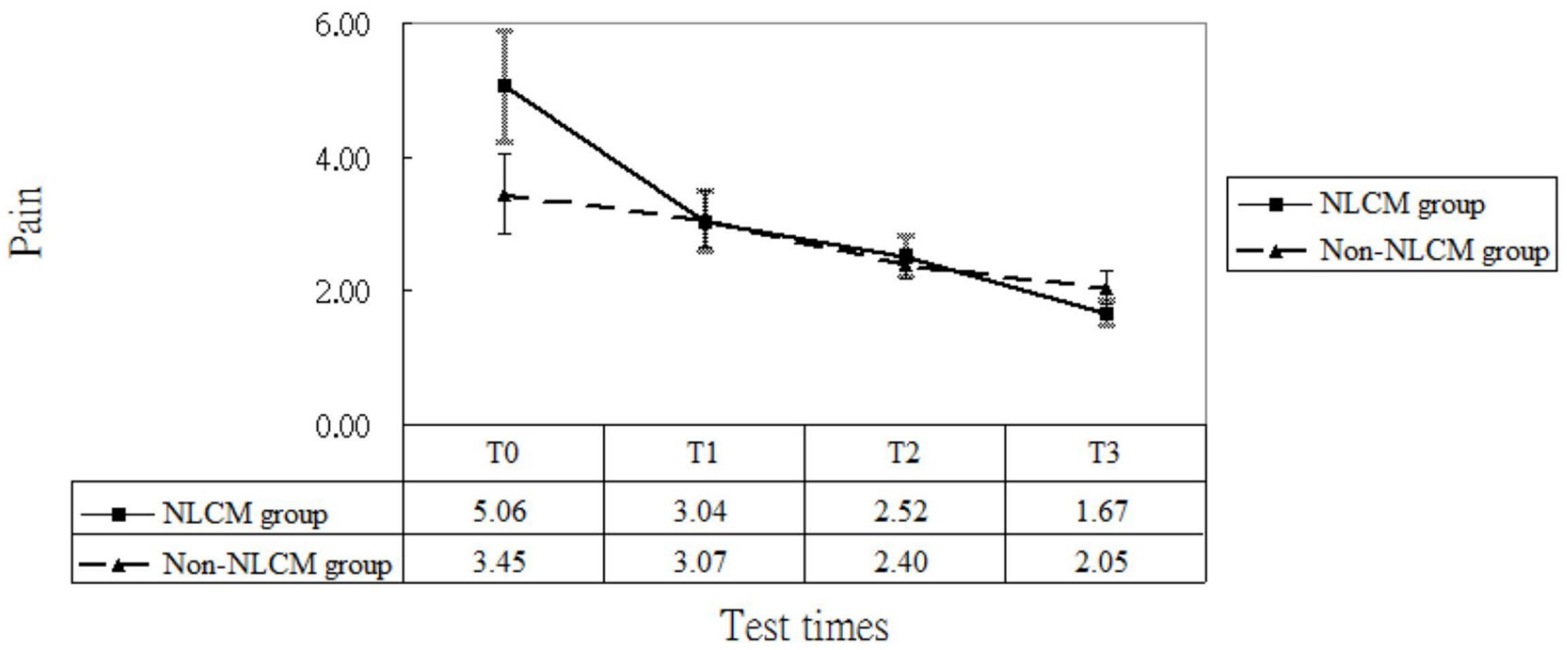
Pain levels of patients in the NLCM and the control groups.

As to pain, the GEE model indicated a difference at T1 and T3, which implied that a maturation effect might occur regardless of the use of intervention employed. After adjustment for age, DAS 28, disease duration, inherent pain level, and maturation effects, we found that NLCM was helpful in reducing pain for RA patients during the study timeframe as compared with the control group, yielding statistical differences of *β* = −1.29 (T1), *β* = −1.49 (T2), and *β* = −1.99 (T3) (Table 2). The change in pain levels between the two groups is displayed in Figure 3.

**Figure 4:**
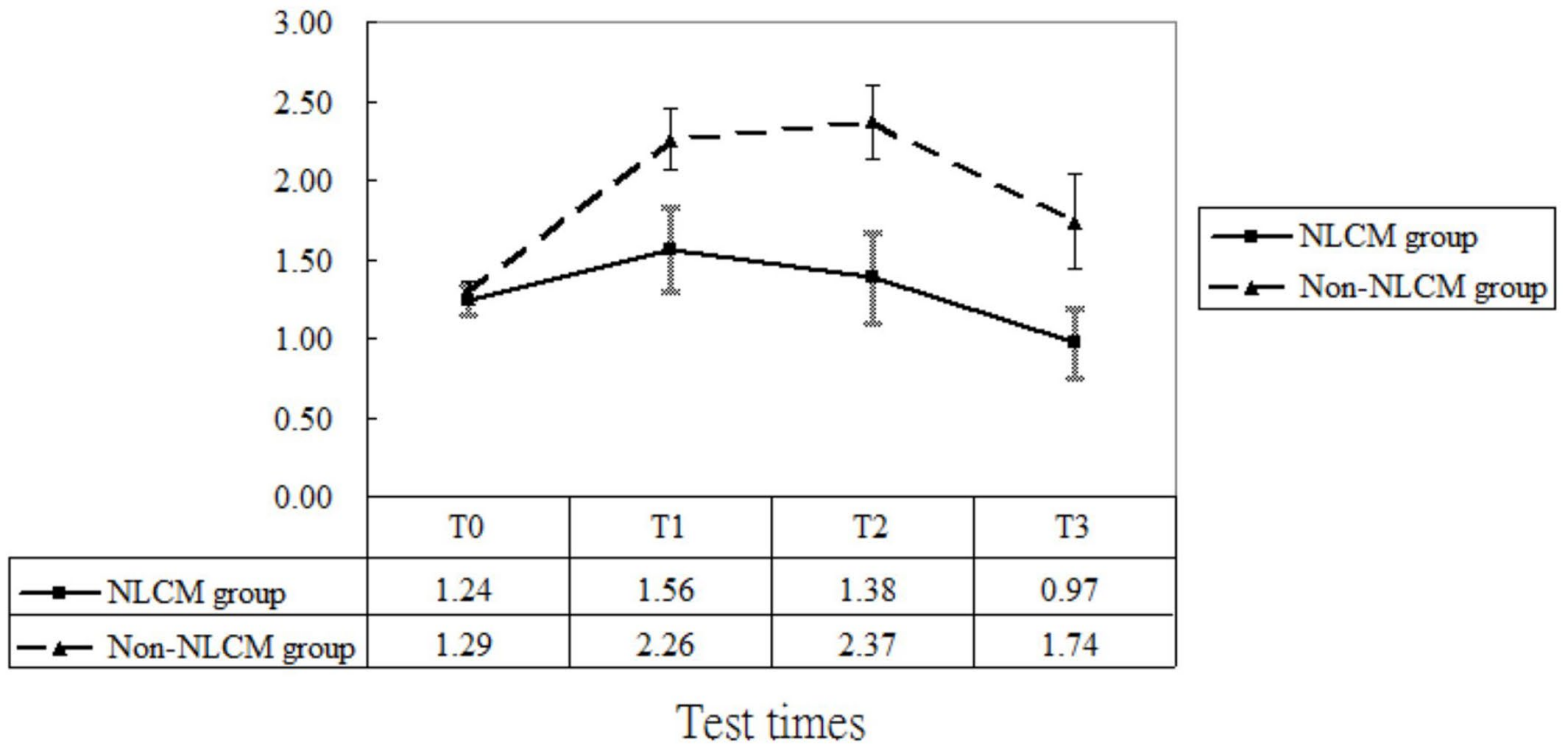
C-reactive protein levels of patients in the NLCM and the control groups.

Regarding CRP, maturation effects were detected at T2 and T3 since CRP levels measured at both T2 and T3 were greater than those measured at T0 (both *p* < 0.05, Table 2). Additionally, the baseline inflammatory status was similar for the two groups (*p* = 0.25, Table 2). After adjusting for the initial differences in age, DAS 28, disease duration, and maturation effects, the mean value of CRP in the NLCM group was found to be lower than in the non-NLCM group at both T1 (*β* = −0.66; *p =* 0.04), T2 (*β* = −0.94; *p =* 0.01), and T3 (*β* = −0.72; *p =* 0.03) (Table 2). These benefits were still detected for 2 years after the NLCM program (Figure 4).

Furthermore, to minimize the baseline imbalances in this comparative study, we carried out one sensitivity analysis where each selected NLCM case was randomly matched to one control without NLCM use via the propensity score matching (34). The propensity score was calculated using logistic regression derived from patients’ demographics and baseline comorbidities at enrollment. Thereafter, a total of 26 NLCM users and 26 control users were included after propensity score matching and no conspicuous differences were found between the two groups, indicating the matched intervention and comparison groups were comparable in terms of baseline characteristics. The reanalysis based on the GEE procedure indicated that NLCM was still significantly related to reductions in levels of pain (T1 = −2.54, *p* < 0.01; T2 = −2.20, *p* = 0.03; T3 = −1.92, *p* = 0.04), fatigue (T1 = −1.27, *p* = 0.02; T2 = −1.31, *p* = 0.01; T3 = −1.25, *p* < 0.01), and CRP (T1 = −0.36, *p* = 0.03; T2 = −0.51, *p* = 0.01; T3 = -0.31, *p* = 0.02), among the enrollees.

## Discussion

While the effects of NLCM have been recognized, few studies have directly examined this association in persons with RA, especially the long-term effects of NLCM on the reduction of fatigue, pain, and intrinsic inflammation. The GEE model used in this study provided further control of participants’ attributes at baseline and temporal maturation effect, enabling us to evaluate the effects of NLCM more precisely. As compared to the participants in the non-NLCM group, we discovered that levels of fatigue, pain, and CRP decreased more in the NLCM group, which implied that the implementation of the 6-month NLCM program into routine care may indeed bring benefits for RA patients. The beneficial impacts herein were consistent with earlier reports (17, 18, 24, 35).

Notably, the findings of our study further supported that the beneficial effects could be maintained for 2 years after the completion of NLCM. We inferred that the implementation by a nursing case manager in the form of one-on-one consultation/education and regular follow-up were the keys. It has been suggested that continuing education activities contributed to better clinical manifestation in chronic disease patients (13). Unlike the traditional care approach from one direction, a highly interactive approach and colored images utilized in the NLCM might be more beneficial in engaging patients in the health information they are learning and assisting them in making appropriate plans for disease management, thereby increasing their at-home self-care skills to diminish the disturbances caused by the RA symptoms. In addition, regular follow-ups may ensure the long-term compliance of the patients. Including fitting physical activity, a key component of NLCM used in this study, may account for the beneficial effect reported herein. A growing body of research findings has indicated that reasonable intermediate exercise loads may decrease leptin levels in serum, which is a well-known adipocytokine that can elevate mRNA and protein expression of inflammatory precursor substances, such as tumor necrosis factor-α (TNF-α) (36). An animal study reported a positive correlation between levels of leptin and interleukin-6 (IL-6) (37). Moreover, the secretions of IL-6 and TNF-α played a role in the development of fatigue in both autoimmune and non-autoimmune diseases via neuroinflammation and neuroprogressive changes (38, 39). Altogether, we propose that early inclusion of a fitting exercise program to the routine pharmacological therapy, as well as prolonging its use, may serve to psychologically benefit RA patients.

The findings of the present study revealed a difference in CRP levels between the NLCM and non-NLCM groups. Since no relevant studies were found to examine the long-term impact of NLCM on CRP in these patients, a direct comparison with earlier studies is impossible. We speculated that direct participation in NLCM programs insensibly contributes to increased socialization and individual self-efficacy, which in turn may assist patients in mitigating the physical and emotional burden of the illness. The concept of self-efficacy has been proven to significantly influence behavior changes that buffer the potential negative effects of various diseases. For example, a recent cross-sectional study by Hladek and colleagues found an association between coping-associated self-efficacy and low serum IL-6 and TNF-α in senior adults (40). These physiologic mediators are known to play indispensable roles in synthesizing and secreting CRP (41). Despite this preliminary evidence, the underlying mechanism of how NLCM alleviates CRP is not well understood, which implies that future large-scale studies to verify the effect of NLCM against inflammatory responses reported herein should be undertaken.

GEE, an extension of the GLM procedure, could model a known function of the marginal expectation of the dependent variable as a linear function of explanatory variables. On top of that, this study was the first to investigate the relation between NLCM and changes in pain, fatigue, and systemic inflammation in RA patients through a long-term follow-up perspective, enabling authors to cautiously shed light on NLCM impacts. Notwithstanding the foregoing issues, this study may be affected by some limitations. First, the sampled participants were selected from a single hospital in Taiwan and accordingly, the generalization of study results may be limited. Second, the subjective scale was used to measure pain or fatigue, so further studies employing more objective measures of psychological change are warranted. Third, the experimental group in this study comprised patients who agreed to take part in the program. Thus, willingness to participate might bias the results of this study. To address this issue, we set objective criteria to balance the baseline differences between the two groups through the evaluation of demographic and disease characteristics, as shown in Table 1. Furthermore, we capitalized on the GEE model to control for possible baseline differences between the two groups (if any) and further consider potential maturation effects. Additionally, the initial descriptive analysis suggested that NLCM users indeed exhibited poorer manifestations than did non-NLCM users, yet they displayed substantial reductions in pain, fatigue, and CRP than did the non-users, implying that the present findings are likely to underestimate, rather than overestimate, the effects of NLCM. Taken together, we concluded that baseline differences between the two groups, in all likelihood, did not distort the present findings. Fourth, even though we used the GEE model to control for baseline differences between the two groups, the application of an observational design herein may still be affected by potential confounders that were not included in the models (33). We conducted a sensitivity analysis by utilizing the propensity score with one-to-one matching to reduce the imbalance of the characteristics between the two groups (34). The reanalysis based on the GEE procedure indicated that NLCM was still related to reductions in levels of pain, fatigue, and CRP, suggesting that the baseline imbalance did not appreciably impact the relationship reported herein. To lend further credence to the present findings, future prospective randomized trials are needed to overcome the experimental weaknesses of this study via employing psychometrically sound measurements, which would allow for more efficient approaches to disease management of RA.

## Conclusion

On the whole, this study supported the idea that adding the NLCM to conventional care can ameliorate the distressing symptoms and systemic inflammation of RA patients. Findings demonstrated that participants in the NLCM group experienced lower levels of pain, fatigue, and CRP after the intervention than their control counterparts. Notably, we observed that these beneficial effects were maintained for 2 years after the completion of NCLM. The findings of this study may be a reference in facilitating the implementation of the NLCM program among patients with newly diagnosed RA for long-term survival benefits. Due to the potential drawbacks regarding recruitment strategy and data collection, the effects of NLCM still must be further elucidated via well-designed, long-term randomized controlled trials.

## Data availability statement

Data are available upon reasonable request. The data used and/or analyzed during the current study are available from the corresponding authors only on academic research request.

## Ethics statement

The studies involving humans were approved by Institutional Review Board and the Ethics Committee of Buddhist Dalin Tzu Chi Hospital No. B11004003. The studies were conducted in accordance with the local legislation and institutional requirements. The participants provided their written informed consent to participate in this study.

## Author contributions

W-CC: Formal analysis, Investigation, Validation, Writing – review & editing, Writing – original draft. HL: Methodology, Writing – review & editing. H-LH: Data curation, Investigation, Writing – original draft. H-HL: Conceptualization, Investigation, Validation, Writing – original draft. M-CLu: Data curation, Project administration, Writing – original draft. M-CLi: Conceptualization, Investigation, Writing – original draft. W-JC: Formal analysis, Funding acquisition, Project administration, Validation, Writing – original draft. T-YT: Conceptualization, Formal analysis, Methodology, Project administration, Validation, Writing – original draft, Writing – review & editing.
